# Sarcopenia is associated with worse surgical complications but not relapse-free survival and overall survival in patients with retroperitoneal liposarcoma

**DOI:** 10.1186/s12957-022-02846-1

**Published:** 2022-12-20

**Authors:** Mingkun Zhao, Minzhi Lv, Yuan Fang, Aobo Zhuang, Qian Wu, Hanxing Tong, Weiqi Lu, Yong Zhang

**Affiliations:** 1 Department of General Surgery, Shanghai Public Health Clinical Center, Fudan University, Shanghai, China; 2 Department of Biostatistics, Zhongshan Hospital, Fudan University, Shanghai, China; 3 Department of General Surgery, Zhongshan Hospital, Fudan University, Shanghai, China

**Keywords:** Retroperitoneal liposarcoma, Sarcopenia, Surgery complication, Relapse-free survival, Overall survival

## Abstract

**Objective:**

This investigation aimed to explore the relationship between sarcopenia and severe postoperative complications, relapse-free survival (RFS), and overall survival (OS) in patients with retroperitoneal liposarcoma (RLPS).

**Material and methods:**

This retrospective study included 72 RLPS patients (47 men, 25 women; mean age, 57.49 years, SD 10.92) who had abdominal CT exams. Clinical information was recorded, including RLPS characteristics (histologic subtypes, grade, size), laboratory assessment (ALB, PALB, A/G, Hb, SCr), relapse-free survival, overall survival, and postoperative complications. The relationships between those variables and RFS and OS were analyzed using Cox proportional hazard models.

**Results:**

There were 8 severe postoperative complications (Clavien-Dindo grade > 2). The chi-square test showed sarcopenia was associated with severe postoperative complications (*P* = 0.011). In multivariate analysis, sarcopenia was not associated with relapse-free survival (*P* = 0.574) and overall survival (*P* = 0.578).

**Conclusions:**

Sarcopenia predicts worse surgical complications but does not affect relapse-free survival and overall survival.

## Summary

This study retrospectively collected data from 72 patients with liposarcoma after en bloc compartment resection for primary RLPS. ImageJ software determined the skeletal muscle index (SMI) diagnostic threshold for defining sarcopenia. Cox regression analyses were performed to determine the independent risk factors for overall survival (OS) and relapse-free survival (RFS).

## Introduction

Retroperitoneal soft tissue sarcomas (RSTS) account for 0.15% of all malignant tumors and approximately 15% of soft tissue sarcomas. Meanwhile, retroperitoneal liposarcoma (RLPS), accounting for 20% of all soft tissue tumors, is the most common retroperitoneal soft tissue sarcoma [1]. Wide local excision with clear surgical margins remains the primary treatment mode for the cure [2]. Recent studies have recognized that CT-derived muscle size correlates with overall survival. Sarcopenia is caused by inactivity, disease, cachexia, malnutrition, and advanced aging, which eventually develops into loss of skeletal muscle mass and decreased functional strength including decreased functional independence and difficulties with ambulation and activities of daily living. Sarcopenia is recognized as an independent risk factor for postoperative complications and mortality in a variety of patients with or without surgery [3].

Because routine CT scans are easily accessed and obtained, the skeletal muscle index (SMI) and psoas muscle index (PMI) is measured to determine sarcopenia. Moreover, body composition parameters, like SMI and PMI, can influence clinical risk assessment and decision-making in different cancer types. For instance, sarcopenia was shown to be an independent prognostic factor for shorter OS in gastric cancer and colorectal cancer, as well as inferior relapse-free survival (RFS) [4, 5].

However, some publications explored the relationship between sarcopenia and clinical outcomes among patients with sarcoma; one reported that there was no relationship between psoas cross-sectional area and clinical outcomes after soft tissue resection [6]. The others demonstrated that sarcopenia was independently associated with poorer long-term prognosis in sarcoma [3, 7–11], and lower psoas muscle attenuation was associated with higher mortality [8, 12]. The advanced studies showed that sarcopenia is associated with prognostic parameters in advanced or metastatic STS and first-line therapy response [13]. Therefore, body composition parameters should be deeply investigated to evaluate the next step of treatment, which needs further prognostication systems to avoid over-or undertreatment as soon as possible.

To our knowledge, sarcopenia has not been evaluated as a postoperative relapse marker for especially liposarcoma. Therefore, this study aimed to explore the influence of preoperative sarcopenia and nutritional status on relapse and survival time. To undertake this, we analyzed a large, single-institution cohort of patients with primarily diagnosed RLPS, carefully assessed for preoperative sarcopenia and nutritional status, and followed up.

## Material and method

This series included all consecutive patients with primary localized RPS who underwent resection between January 2012 and December 2020 by a high-volume sarcoma center at the Zhongshan Hospital and Shanghai Public Health Clinical Center. We collected 72 patients with retroperitoneal liposarcoma who received enhanced CT or MRI and had complete clinical data. The collection of clinical data included: gender, age, body mass index (BMI), albumin (ALB), pre-albumin (PALB), albumin-to-globulin (A/G), hemoglobin (Hb), serum creatinine (SCr), preoperative symptoms, histologic subtypes, FNCLCC (the Fédération Nationale des Centres de Lutte Contre le Cancer) grade, tumor burden, combined-organ resection, operation duration, estimated blood loss, blood transfusion, ICU stays, postoperative hospital stays, postoperative complications. All retroperitoneal liposarcoma histotypes were diagnosed by the pathologist. An abdominopelvic CT or MRI scan of diagnostic quality within 14 days before operation was required from the PACS system.

All the cases were first-time diagnosed with LPS and performed R0 excision. Ethical approval was obtained for this retrospective study and conducted in compliance with the Declaration of Helsinki. The evaluation included severe postoperative complications, relapse-free survival (RFS), and overall survival (OS) in patients. The definition of RFS was the time from diagnosis to relapse, death, or the study termination date, while OS was the time from diagnosis to the time of death or the study termination date.

### Outcome measures

The same axial CT image at the L3 pedicle level was used to obtain fat and muscle measurements. Total cross‐sectional skeletal and psoas muscle areas were measured using image processing software (ImageJ, 1.53c; National Institutes of Health, USA http://imagej.nih.gov/ij). The following were measured: (I) L3 skeletal muscle area (cm^2^); (II) L3 psoas muscle area; (III) L3 fat area (cm^2^) (by measuring the subcutaneous adipose tissue area). Sarcopenia was defined as SMI < 43.13 cm^2^/m^2^ for men and < 37.81 cm^2^/m^2^ for women [14].

### Statistical analysis

Numerical variables were shown by mean ± SD, and categorized variables were summarized by absolute frequencies. Continuous variables were compared by Student’s *t*-test, and categorized variables were compared by the *χ*^2^ test (or Fisher’s exact test as required) across two groups (with sarcopenia and non-sarcopenia). Univariable and multivariable analyses for RFS and OS were performed using the Cox proportional hazards model to evaluate the effects of each variable. Variables with *P*-value < 0.1 in the univariate analysis or variables identified by clinical experience were further included in the multivariate analysis. Results were considered significant at *P* < 0.05. Descriptive statistics and analyses were obtained using SPSS 26.0 (IBM corp., Armonk, USA).

## Results

Patient baseline characteristics are shown in Table 1. All patients were divided into two groups based on whether they had preoperative sarcopenia. Whether patients had sarcopenia was not significantly associated with sex, age, ALB, PALB, A/G, SCr, fat area, preoperative symptoms, histologic subtypes, FNCLCC grade, tumor burden, combined-organ resection, operation duration, estimated blood loss, blood transfusion, ICU stays, postoperative hospital stays. However, there was a significant association between sarcopenia and BMI (*P*-value = 0.007) and hemoglobin (*P*-value = 0.033). The Chi-square test showed no association between complications and sarcopenia (*P*-value = 0.362). However, there was statistically significant (*P*-value = 0.011) when comparing severe postoperative complications (Clavien-Dindo grade > 2) with sarcopenia. In detail, severe postoperative complications occurred in 8/42(19.0%) patients with sarcopenia while in 0/30 (0%) patients without sarcopenia. RLPS patients with sarcopenia were more likely to have serious complications after surgery, resulting in severe perioperative recovery.

**Table 1:** Surgical characteristics and pre-operative laboratory parameters in 72 patients with primary retroperitoneal liposarcoma

Variables	Total *N* = 72	Sarcopenia	Non-sarcopenia	*P* value
Gender				0.086
Male	47	24	23	
Female	25	18	7	
Age (years) (mean ± SD)	57.49 ± 10.92	59.13 ± 11.64	55.3 ± 9.64	0.148
Symptoms				0.905
Yes	33	19	14	
No	39	23	16	
Histologic subtypes				0.331
Well-differentiated (WDLPS)	47	25	22	
Myxoid/round cell (MLPS)	6	4	2	
Dedifferentiated (DDLPS)	18	13	5	
Pleomorphic (PLPS)	1	0	1	
FNCLCC				0.334
Grade1	31	19	12	
Grade2	27	13	14	
Grade3	14	10	4	
Tumor burden, cm median (IQR)	20, 15–29.5	20, 13–29	20, 15–30	0.402
Combined-organ resection				0.371
Yes	54	32	22	
No	18	10	8	
Operation duration, hours median (IQR)	4.0, 3.0–5.0	4.0, 3.0–5.0	4.0, 2.0–5.0	0.959
Estimated blood loss, mL median (IQR)	400, 200–700	500, 100–825	300, 200–500	0.160
Blood transfusion				0.213
Yes	20	14	6	
No	52	28	24	
ICU stay				0.468
Yes	49	30	19	
No	23	12	11	
Complications				0.362
Yes	26	17	9	
No	46	25	21	
Clavien-Dindo classification				0.011*
≤ 2	64	34	30	
> 2	8	8	0	
Postoperative hospital stays, days median (IQR)	15, 12–22.75	16, 13–24	14, 9–21	0.093
BMI (mean ± SD)	23.48 ± 3.78	22.48 ± 3.79	24.9 ± 3.35	0.007*
ALB, g/L (mean ± SD)	38.51 ± 5.77	38.44 ± 5.74	38.61 ± 5.91	0.904
PALB, g/L (mean ± SD)	0.21 ± 0.08	0.20 ± 0.08	0.22 ± 0.08	0.375
A/G (mean ± SD)	1.46 ± 0.39	1.47 ± 0.39	1.46 ± 0.39	0.908
Hb, g/L (mean ± SD)	124.42 ± 17.01	120.76 ± 16.35	129.43 ± 16.88	0.033*
SCr,μmol/L (mean ± SD)	74.3 ± 18.15	74.09 ± 20.27	74.61 ± 15.07	0.907
SMA, cm^2^ (mean ± SD)	111.81 ± 28.81	95.47 ± 17.39	134.67 ± 26.02	0.000
SMI (mean ± SD)	40.64 ± 8.85	35.03 ± 4.79	48.5 ± 7.05	0.000
PMA, cm^2^ (mean ± SD)	14.23 ± 5.06	12.28 ± 3.66	16.95 ± 5.54	0.000
PMI (mean ± SD)	5.15 ± 1.63	4.49 ± 1.14	6.08 ± 1.77	0.000
Fat area, cm^2^ (mean ± SD)	78.24 ± 46.7	76.26 ± 44.48	80.56 ± 49.86	0.719

*BMI* body mass index, *ALB* albumin, *PALB* pre-albumin, *A/G* albumin globulin, *Hb* hemoglobin, *SCr* serum creatinine, *SMA* skeletal muscle area, *SMI* skeletal muscle index, *PMA* psoas muscle area, *PMI* psoas muscle index

Of all 72 patients with RLPS who met the criteria, the median follow-up time for the survivors (*n* = 72) was 50.5 months (IQR 34.75–66.25). In all patients, 40 (55.5%) suffered from relapse. The median RFS was 35 months (IQR 20.75–60.5). Furthermore, the 2- and 5-year RFS rates were 34.0% and 21.9%, respectively. Those patients had 12 (13.1%) deaths, with a median survival time of 50.5 months.

### Prognostic factors for disease relapse

**Fig. 1:**
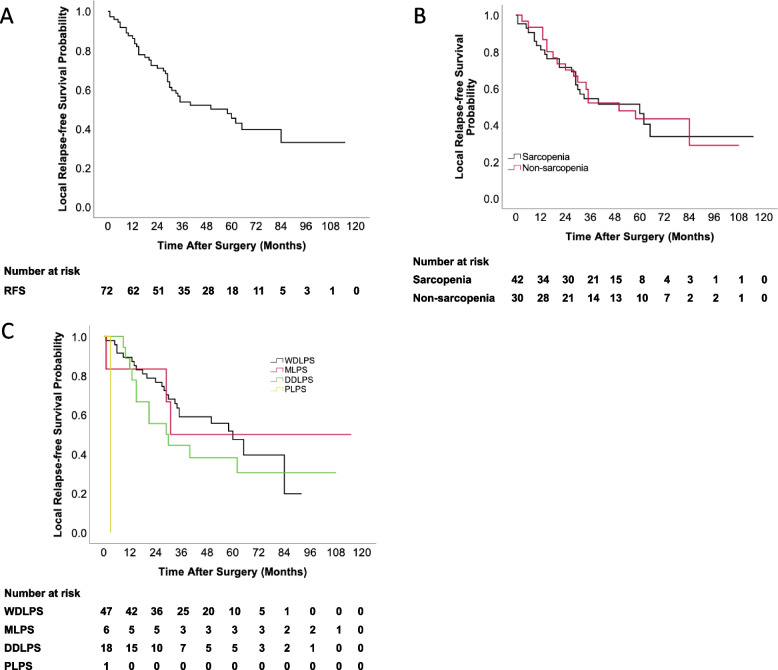
Local relapse-free survival in patients by **A** all patients, **B** sarcopenia, and **C** histologic subtypes

Table 2 showed a univariate and multivariate analysis of important prognostic factors for relapse. In the univariable analyses, sarcopenia did not show a significant association with relapse-free survival (*P*-value = 0.574). However, higher SCr was associated with an impaired relapse-free survival (HR 1.024, 95% CI: 1.004–1.043, *P* = 0.017). Compared to WDLPS, we observed that DDLPS- and PLPS-identified patients got a higher risk of relapse. In contrast, MLPS-identified patients got a lower risk of relapse (Table 2). Symptoms (HR 1.918, 95% CI: 1.025–3.589, *P*-value = 0.042), and postoperative hospital stays (HR 1.02, 95% CI: 1.005–1.035 *P*-value = 0.007) were also related to RFS. FNCLCC grade (*P*-value = 0.158) was also included in the multifactorial analysis, as it was reported to be an important risk factor for RFS [15, 16]. In multivariate analysis, higher SCr level (HR 1.036, 95% CI: 1.013–1.060, *P*-value = 0.002) and histologic subtypes (*P*-value = 0.018) were independent risk factors for RFS. The RFS rates were 86% at 1 year, 71% at 2 years, and 25% at 5 years (Fig. 1). There was no significant difference between sarcopenia and postoperative recurrence (*p* = 0.574) (Fig. 1). Histologic subtype was significantly associated with RFS (*p* = 0.018). The probability free of recurrence at 3 years for WDLPS, DDLPS, MLPS, and PLPS was 53%, 39%, 50%, and 0%, respectively (Fig. 1).

**Table 2:** Univariable and multivariable analyses to determine independent predictors of relapse-free survival of primary retroperitoneal liposarcoma

Variables	Univariate analysis		Multivariate analysis	
	Hazard ratio (95% CI)	*P*-value	Hazard ratio (95% CI)	*P*-value
Gender (male vs. female)	1.341 (0.691–2.603)	0.386	0.406 (0.155–1.063)	0.067
Age	1.013 (0.985–1.043)	0.360	1.018 (0.987–1.050)	0.258
BMI	0.961 (0.89–1.038)	0.315		
ALB	1.015 (0.956–1.078)	0.616		
PALB	0.382 (0.007–21.583)	0.640		
A/G	1.002 (0.441–2.279)	0.995		
Hb	0.998 (0.979–1.017)	0.808		
SCr	1.024 (1.004–1.043)	0.017*	1.036 (1.013–1.060)	0.002*
SMA	1 (0.99–1.01)	0.989		
SMI	0.992 (0.96–1.025)	0.632		
PMA	0.991 (0.935–1.049)	0.746		
PMI	0.94 (0.784–1.128)	0.507		
Fat area	1 (0.993–1.007)	0.960		
Sarcopenia (yes vs. no)	1.061 (0.566–1.989)	0.853	0.804 (0.376–1.719)	0.574
Symptoms (yes vs. no)	1.918 (1.025–3.589)	0.042*	1.300 (0.653–2.588)	0.455
Histologic subtypes		0.021*		0.018*
MLPS vs. WDLPS	0.822 (0.244–2.767)		0.454 (0.082–2.519)	
DDLPS vs. WDLPS	1.451 (0.722–2.915)		0.553 (0.144–2.120)	
PLPS vs. WDLPS	38.718 (3.462–433.047)		44.905 (3.462–582.423)	
FNCLCC		0.158		0.093
Grade 2 vs. 1	1.749 (0.85–3.601)		2.593 (1.010–6.660)	
Grade 3 vs. 1	2.074 (0.918–4.688)		5.006 (0.930–26.946)	
Tumor burden	0.991 (0.958–1.024)	0.580		
Combined-organ resection (yes vs. no)	1.418 (0.651–3.088)	0.379		
Operation duration	0.988 (0.781–1.249)	0.918		
Estimated blood loss	1 (1–1)	0.337		
Blood transfusion (yes vs. no)	1.33 (0.672–2.632)	0.413		
ICU stay (yes vs. no)	1.895 (0.924–3.886)	0.081	1.290 (0.535–3.110)	0.57
Postoperative hospital stays	1.02 (1.005–1.035)	0.007	1.013 (0.995–1.031)	0.167
Complication (yes vs. no)	1.303 (0.69–2.46)	0.415		

* means *P*-value < 0.05

### Overall survival analysis

**Fig. 2:**
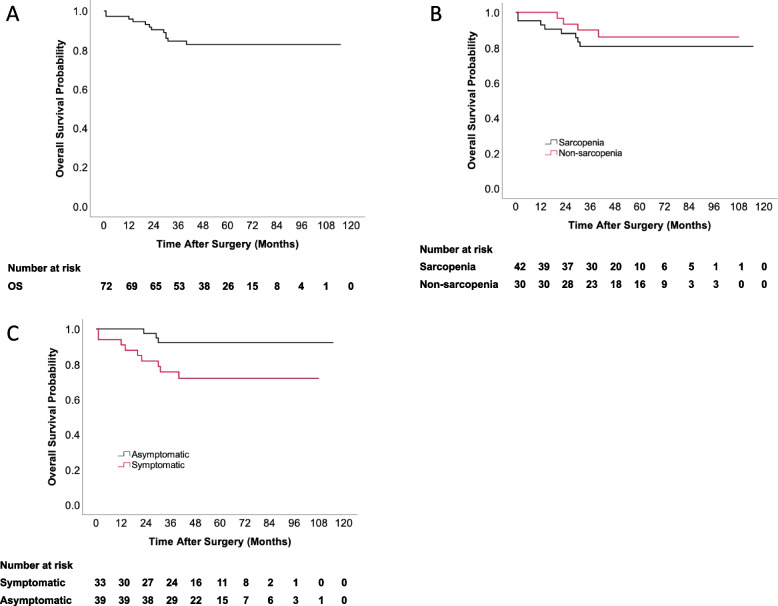
Overall survival in patients by **A** all patients, **B** sarcopenia, and **C** symptoms

The univariate analysis of risk factors for OS was shown in Table 3. Again, sarcopenia had no correlation with overall survival (*P*-value = 0.483). Higher serum creatinine level was associated with an inferior overall survival (HR 1.044, 95% CI: 1.020–1.068 *P* < 0.001). Patients with preoperative symptoms emerged as a strong predictor of overall survival (HR 3.954, 95% CI: 1.070–14.613, *P*-value = 0.039). The postoperative hospital stays (HR 1.024, 95% CI: 1.006–1.041, *P*-value = 0.008) were also associated with the endpoints in this study. The multivariable model showed that sarcopenia did not relate to OS (*P*-value = 0.578), and a higher level of SCr was confirmed as a significant independent prognostic factor for worse overall survival (HR 1.043, 95% CI: 1.011–1.076 *P*-value = 0.008). The preoperative symptoms were also an important independent prognostic factor (HR 4.371, 95% CI: 1.018–18.768, *P*-value = 0.047). The OS rates were 96% at 1 year, 90% at 2 years, and 36% at 5 years (Fig. 2). There was no significant difference between sarcopenia and overall survival (*p* = 0.578) (Fig. 2). Compared with asymptomatic patients at the time of consultation, symptomatic patients had a worse OS (*p* = 0.047) (Fig. 2).

**Table 3:** Univariable and multivariable analyses to determine independent predictors of overall survival of primary retroperitoneal liposarcoma

Variables	Univariate analysis		Multivariate analysis	
	Hazard ratio (95% CI)	*P*-value	Hazard ratio (95% CI)	*P*-value
Gender (male vs. female)	6.342 (0.818–49.159)	0.077	2.034 (0.203–20.361)	0.546
Age	1.020 (0.968–1.076)	0.460	0.996 (0.936–1.06)	0.893
BMI	0.915 (0.785–1.066)	0.256		
ALB	0.994 (0.902–1.095)	0.902		
PALB	2.856 (0.003–2974.716)	0.767		
A/G	0.695 (0.163–2.961)	0.622		
Hb	0.996 (0.965–1.029)	0.814		
SCr	1.044 (1.020–1.068)	< 0.001*	1.043 (1.011–1.076)	0.008*
SMA	1.006 (0.988–1.024)	0.513		
SMI	1.007 (0.947–1.072)	0.820		
PMA	1.012 (0.907–1.129)	0.829		
PMI	0.967 (0.677–1.381)	0.853		
Fat area	0.995 (0.980–1.010)	0.511		
Sarcopenia (yes vs. no)	1.537 (0.463–5.106)	0.483	1.494 (0.363–6.151)	0.578
Symptoms (yes vs. no)	3.954 (1.070–14.613)	0.039*	4.371 (1.018–18.768)	0.047*
Histologic subtypes		0.638		
MLPS vs. WDLPS	1.368 (0.165–11.373)			
DDLPS vs. WDLPS	2.198 (0.671–7.203)			
PLPS vs. WDLPS	0 (0–0)			
FNCLCC		0.150		0.571
Grade 2 vs. 1	1.557 (0.348–6.962)		2.087 (0.384–11.351)	
Grade 3 vs. 1	3.827 (0.914–16.024)		2.37 (0.427–13.159)	
Tumor burden	0.987 (0.928–1.050)	0.676		
Combined-organ resection (yes vs. no)	1.583 (0.347–7.226)	0.553		
Operation duration	1.082 (0.736–1.592)	0.688		
Estimated blood loss	1 (1.000–1.001)	0.398		
Blood transfusion (yes vs. no)	1.928 (0.612–6.075)	0.262		
ICU stay (yes vs. no)	39.955 (0.279–5724.408)	0.145		
Postoperative hospital stays	1.024 (1.006–1.041)	0.008*	1.004 (0.984–1.025)	0.677
Complication (yes vs. no)	1.271 (0.403–4.007)	0.682		

* means *P*-value < 0.05

## Discussion

Identifying patients with different prognoses according to their clinical and pathological conditions is essential to provide individualized treatment and improving the efficacy of liposarcoma treatments. According to the publication of the European Working Group on Sarcopenia in Older People consensus in 2019 (EWGSOP2) [17] and the Asian Working Group for Sarcopenia (AWGS) 2019 [18], sarcopenia is characterized by a low skeletal muscle mass, low muscle strength, and poor low physical performance. Many reports have shown that sarcopenia is closely related to the prognosis of patients with various malignant tumors. [3, 6–11, 13, 19–21] However, different pathological types of sarcomas are not identical in treatment and prognosis, so we conducted a study on the exact pathological type of sarcomas and drew relevant conclusions. Meanwhile, the effect of sarcopenia on the post-surgical outcomes of patients with liposarcoma undergoing en bloc compartment resection has not been reported. This article is the first retrospective study to investigate the association between sarcopenia and a specific pathological type of sarcoma in a homogeneous population of patients with retroperitoneal liposarcoma treated by radical surgery performed in an independent large-volume institution.

Routine CT scans were easy to access, wherein the skeletal muscle index (SMI) is measured for sarcopenia determination. Currently, the cutoff value point of sarcopenia remains controversial mainly because of the differences among different ethnic groups. The previous definition mentioned by Guohao Wu et al.[14] included 6447 samples, and the cutoff value was the lowest sex-specific quartile of SMI at L3 in those patients. Therefore, we chose the cutoff point defined by Guohao Wu et al. (43.13 cm^2^/m^2^ for men and 37.81 cm^2^/m^2^ for women).

The interest in pre- and post-operative nutrition is already recognized. Traditional metabolic and nutritional care of patients undergoing major elective surgery has emphasized preoperative fasting and reintroduction of oral nutrition 3–5 days after surgery [22]. Because the RLS has an invasive tendency and the invasion of the intestine will be seen from time to time as well as combined multiple organ resection will be seen in many RLS patients, which causes a lengthy recovery period for the gastrointestinal function, and then prolonged fasting time and injure the nutritional condition of post-operation patients. That is why evaluating the nourishment level before the operation's implementation is essential. The post-operation nutritional condition is also vital to the recovery of patients. Furthermore, the poor preoperation nutritional condition will also increase the post-operation recovery time and incidence of death and complications [7]. The results of this study showed that patients with sarcopenia had low BMI and hemoglobin levels. There was no association between sarcopenia and postoperative complications, but sarcopenia was associated with severe complications, which are defined as the Clavien-Dindo grade > 2. This means that RLPS patients with sarcopenia are more likely to expose to severe and fatal complications. In previous studies, the results showed that sarcopenia is not an independent predictor of postoperative complications [3]. However, no one compared the association between sarcopenia and severe complications in patients with LPS.

Many reports showed that sarcopenia was related to poor RFS or distant metastasis-free survival (DMFS), such as Extrahepatic Cholangiocarcinoma, Pancreatic Ductal Adenocarcinoma, nasopharyngeal carcinoma, non-small cell lung cancer, and colorectal cancer. [5, 15, 23–25]The mean mechanism for this result might be that sarcopenia is associated with immune senescence and reduces cancer immunity, which induces the reduction of immunity to inflammation. The inflammatory microenvironment is changed, which is involved in carcinogenesis and cancer progression [26, 27]. However, in our study, there was no strong correlation between sarcopenia and the RFS and OS of patients with RLPS. We used Cox proportional hazard model, and univariable and multivariable analyses showed that the higher level of SCr was an independent risk factor for RFS and confirmed as a significant independent prognostic factor for worse overall survival, which is partly similar to previous studies [28, 29]. However, underlying mechanisms for this association in liposarcoma patients have not been elucidated. The possible reason is that creatine and Phosphocreatine are essential energy sources donating ATP. The serum creatinine as a waste product of the creatine metabolism for donating ATP might increase during cancer progression. Therefore, with highly active tumors, serum creatinine levels might be higher [30]. The histologic subtype was an independent risk factor for RFS, and the preoperative symptom was a significant independent prognostic factor of OS.

Speculations on why there was no association between sarcopenia and the RFS and OS in RLPS are listed below. First, the pathological types of sarcomas are numerous and complicated. In previous studies, which demonstrated that sarcopenia was independently associated with poorer long-term prognosis in sarcoma, the pathological types of sarcomas were not stratified and analyzed, and they could not answer the specific influence of sarcopenia on a particular kind of pathological type of sarcoma. Second, the number of patients included in this study was limited, and the follow-up time was not long enough to see the outcome of every patient. Third, the biological origin and inflammatory microenvironment may differ in RLPS than in other kinds of carcinoma, so sarcopenia impacts fewer in RLPS than in different carcinomas. Although sarcopenia was predicted to cause worse surgical complications, there was no association between sarcopenia and relapse-free survival and overall survival in RLPS, which was consistent with the previous study [31]. Therefore, further research should be done with a larger sample, a longer follow-up, and a deeper investigation of molecular and biological mechanisms of sarcopenia in LPS. Nevertheless, this is the first study to investigate the potential impact of sarcopenia on the long-term outcomes of patients with RLS. If the result is still widely applicable in an enlarged sample, then we can be reassured that there is no long-term oncologic penalty to be paid for sarcopenia in LPS.

Although RLPS is a sporadic disease, we collected all accessible cases. Nevertheless, this study still had some limitations. First, most patients with liposarcoma were primarily diagnosed, and the local recurrent cases were excluded, so the number of patients was limited, which may cause bias. Second, this study was a retrospective study conducted in a single institution and included the Asian population only; therefore, further prospective studies and data from Western countries should be investigated. Third, due to the relatively few cases of stratified analysis of pathological subtypes, we did not identify sarcopenia related to liposarcoma subtypes, which might affect the prognostic value of sarcopenia for liposarcoma. Therefore, we need a larger sample size to determine and verify the prognostic effect of sarcopenia on different pathological types of RLPS. Anyway, to our knowledge, this is the first study to explore the impact of sarcopenia on the short-term and long-term outcomes in patients with RLPS by using data from a single large-volume institution, thus providing a reference for future clinical trials.

## Conclusions

In this cohort, for first-time patients diagnosed with RLPS and performed R0 resection, sarcopenia was not associated with relapse-free survival and overall survival but predicted worse surgical complications. A higher SCr level is associated with worse RFS and OS. At the same time, histologic subtypes of RLPS are an independent risk factor for RFS, and the preoperative symptoms are a significant independent prognostic factor for OS.

## Data Availability

The datasets used and analyzed during the current study are available from the corresponding author on reasonable request.
